# Bibliographical Notices

**Published:** 1838-10-10

**Authors:** 


### BIBLIOGRAPHICAL NOTICES.

*Homœopathic Practice of Medicine.* By Jacob Jeanes, M. D. Philad., 1838. 8vo. pp. 38.

Of late years, there have been two distinct classes of medical works. One has been characterized by a rigid observation of disease, and by a critical examination of the powers of thereapeutics, bordering at times on scepticism. The other takes quite an opposite direction: the natural course of disease is overlooked, and the most exaggerated importance attached to the influence of remedies, however various they may be in their nature, and however much they may differ amongst themselves, as to the mode of their administration. For specimens of the first class of works, we have not far to look. Our readers must be more or less acquainted with the thorough and systematized observation of many of the later French writers, and the scarcely less accurate works of some of the physicians of Great Britain, particularly of those of the Dublin school. Works of this character have been thus far most useful in pointing out the symptoms of disease, and their relation with internal lesions, and in showing the natural tendency, both of acute and chronic diseases to terminate in recovery or in death, under ordinary circumstances. That is, most acute diseases are now known to tend towards recovery under all circumstances not excessively unfavourable; while, on the other hand, the large proportion of chronic diseases, and a few acute affections, do tend towards death, even under conditions apparently most favourable to the recovery of the patient. Now, this distinction, which had been already pointed out in many admirable treatises of the ancient writers, has been more thoroughly appreciated since observation is more systematically directed to the study of diseases.

In therapeutics, the influence of this class of works has been operating more silently, and has been from time to time checked by numerous obstacles, arising partly from the inherent difficulties of the subject, and partly from our natural unwillingness to acknowledge the failure of our attempts to control disease. Nevertheless, physicians have certainly begun to derive much advantage, even as to therapeutics, from a correct knowledge of pathology, and from the application of sound philosophical observation to the effects of remedies, in diseases of which the natural termination is known, or in removing symptoms which must necessarily incommode the patient, and which may endanger life. Now, the advance of therapeutics, speaking of it as a science, is necessarily gradual, and such continued efforts are required to produce a very slender accession to our stock of knowledge, that many minds are dissatisfied with this slow progress, and seek to resolve the difficulties by some general system which is applicable to all cases, and contains the essence of all that is worthy of being known in medicine.

The slow progress made in therapeutics, and the difficulties which still prevent the treatment of disease from being placed upon the same scientific basis, as its diagnosis, have given rise to the numerous systems which for a time have found their advocates. However fanciful or absurd a new system of practice may be, it will always be favourably received amongst a portion of the public; for, under all circumstances, the majority of acute affections terminate in recovery, and the greater part of chronic diseases are so slow in their course, as readily to deceive any one who is not perfectly conversant with their pathology. Now, if this be true, as applied to various systems of medical practice of an active kind, which, if unskilfully directed, is positively injurious, we can readily conceive how very difficult it must be for many minds to understand how an acute disease can get well, or a chronic affection not become much worse under a simply expectant practice. In either case, the relief felt by the patient, on the decline or remission of the disease, is referred to the action of a dose of a remedy too small for us even to conceive of its existence, while the natural course of the affection is wholly overlooked.

As long as a disease which is treated by the simple withdrawal of injurious agents either tends naturally to recovery or is in its nature incurable, little harm is done to the patient. We will even go much farther, and maintain that some positive advantage must, for a time, result from a practice which, however insignificant it may be, at least has the advantage of soothing the impatience of the sick, and of inducing them quietly to wait for the gradual working of the human system, which is, in most instances, sufficient to relieve it of disease.

In an immense proportion of cases, however, the imagination of the patient can do but little service, and nature is either totally unable to arrest the disease, or brings about a favourable termination, at the expense of much suffering and inconvenience to the patient. In these cases medicine is most obviously useful, and undoubtedly prevents unnecessary waste of life, while it relieves the patient from much of the suffering which is always caused by disease. The physician is then called upon to interfere actively, and is totally unjustifiable in withholding remedies which may quickly relieve his patient of an acute disease, or, which is still more important, may prevent that acute disease from terminating in incurable organic lesions.

We have witnessed within the last year two cases, treated by the Homœopathic method, which precisely illustrate what we have just stated. One of these cases, was apoplexy with complete hemiplegia; there was no vascular excitement,and the patient was a feeble and emaciated old man. He had taken the powders prescribed for him, and had certainly done just as well as under any mild plan of practice, and undoubtedly better than he would have done, if debilitating measures had been used. The case was of course incurable, and in the stage, at which the patient then was, no indication for an efficient treatment presented itself.

The other case was of a very different nature; it was a patient who was ill with dysentery and intermittent. The disease had been allowed to go on until the patient became delirious, and was rapidly sinking. Quinine and opium quickly arrested both the fever and the affection of the bowels. In both these cases, there is no doubt that the disease pursued its natural course; in one case, however, all treatment would have proved inefficacious; and in the other, injustice was certainly done to the patient by withholding remedies which were perfectly adapted to the cure of the disease.

In most cases of disease, physicians feel themselves obliged to use the remedies which seem most adapted to its cure; hence it is very rare that we observe a case of disease which has not been modified by the action of remedies. There is no doubt, that science would be greatly advanced by a collection of carefully observed cases which were either left to themselves, or which have been treated by moral influences alone; now, those amongst the Homœopathic physicians who are educated physicians, might certainly render much service to the science of medicine, if they would publish a collection of cases of disease treated according to their method. The moral reasons which must prevent a physician, who is not a believer in the fanciful notions of Hahnemann, from adopting a purely expectant practice in most diseases, are of course not applicable to those who are firm believers in the doctrine. We hoped that the work of Dr. Jeanes was written in this spirit, and we should have been highly gratified if we could have availed ourselves of his assistance in studying the natural history of disease as a basis for future therapeutics. If a careful statement had been given of all the cases of a particular disease which were treated by infinitesimal doses, we might have compared the success of such a mode with that obtained from a more efficient treatment. As it is, the work consists chiefly of a compilation from the writings of German Homœopaths,—makes as large demands upon our imagination, and gives us as little material for critical examination.

We are not disposed to cavil at such writings, but we cannot admit their utility unless a statement of the duration and result of diseases is furnished us. We require this kind of proof from the most scrupulous observers, and we reject all pretensions to new methods of cure, which are not supported by the most closely sifted evidence. How much more sceptical must we become, when a whole system is presented to us, which is not only at variance with the observations of authors, but with all ordinary modes of reasoning.

These remarks are, of course, applicable to the Homœopathic system after it was brought to something like its present developement. At its origin it was much nearer the truth, and had not the laws which govern all inductive reasoning been lost sight of, it might have developed some very important therapeutic facts. The earlier works of Hahnemann, will perhaps, occupy us at a future period.

---

HEALTH AND BEAUTY. *An Explanation of the Laws of Growth and Exercise; through which a pleasing contour, symmetry of form, and graceful carriage of the body are acquired, and the common deformities of the spine and chest prevented.* By JOHN BELL, M. D., &c. &c. Philadelphia. Carey & Hart. 1838. 18mo., pp. 253.

THIS volume is intended for the general rather than the professional reader. It has claims upon our notice, from the reputation of the author as well as from the excellence of the design and execution of the work. Dr. Bell is one of the few writers upon popular medicine, who has successfully treated this difficult subject. Resorting neither to charlatanry nor to indelicacy to render his book attractive, he has confined himself to the delivery of a few important hygienic and physiological truths, conveyed in a pleasing style, and very happily illustrated. It would be out of place to notice at length the contents of the work, of which a general idea is conveyed by the title. We shall add only, that we think it entitled to the sanction of the profession, and that it may be safely recommended to the public as a very agreeable and useful publication.

